# Supplementary material for: Ariadne: synthetic long read deconvolution using assembly graphs
Source: Genome Biol. 2023 Aug 28;24:197. doi: 10.1186/s13059-023-03033-5 (PMC10463629; doi:10.1186/s13059-023-03033-5)
Supplement: Supplementary file 1 — Additional file 1. Supplementary tables and figures with an extended comparison of Ariadne and Minerva on a subset of the full MOCK5 10x dataset demonstrating Ariadne’s improvements in deconvolution. [file 13059_2023_3033_MOESM1_ESM.pdf]

Supplementary Materials

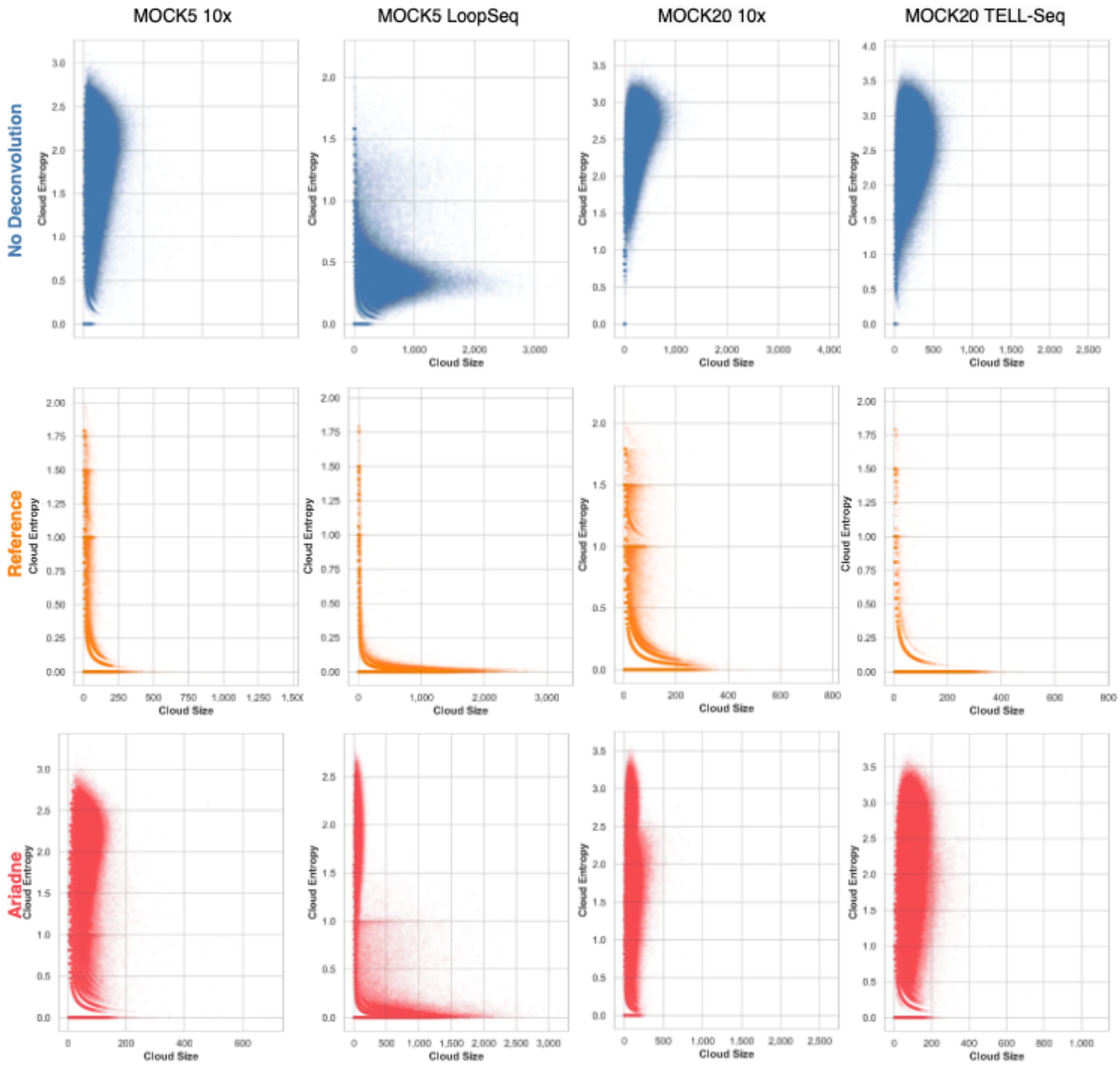

Supplementary Figure 1: Ariadne and reference deconvolution greatly reduced large, high-entropy read clouds and create a large population of read clouds with entropy H = 0. However, the range of entropy remains the same, albeit significantly shifted towards H = 0.

Dataset Name	Num. Reads	Num. Barcoded Reads	Prop. Barcoded Reads	Species	Download	Reference Sequences	Product Sheet
MOCK5 10x	97,491,080	91,101,472	0.93446	Escherichia coli, Enterobacter cloacae, Micrococcus luteus, Pseudomonas fluorescens, Staphylococcus epidermidis	https://s3.us-east-2.amazonaws.com/readclouds/cloudspades_data.tar.gz	https://github.com/lauren-mak/ariadne/tree/spades_3.12.0/reference_seqs	Discontinued
MOCK5 LoopSeq	75,107,814	75,107,814	1	Escherichia coli, Porphyromonas gingivalis, Pseudomonas aeruginosa, Rhodobacter sphaeroides, Streptococcus mutans	https://www.ncbi.nlm.nih.gov/bioproject/PRJNA728470		Discontinued
MOCK20 10x*	100,000,000	94,151,528	0.94152	Streptococcus mutans, Porphyromonas gingivalis, Staphylococcus epidermidis, Escherichia coli, Rhodobacter sphaeroides, Bacillus cereus, Pseudomonas aeruginosa*, Streptococcus agalactiae, Clostridium beijerinckii, Staphylococcus aureus, Acinetobacter baumannii, Neisseria meningitidis, Propionibacterium acnes, Helicobacter pylori, Lactobacillus gasseri, Bacteroides vulgatus, Deinococcus radiodurans, Actinomyces odontolyticus, Bifidobacterium adolescentis, Enterococcus faecalis	https://s3.us-east-2.amazonaws.com/readclouds/cloudspades_data.tar.gz		https://www.atcc.org/products/all/MSA-1003.aspx
MOCK20 TELLSeq*	100,000,000	100,000,000	1	Same as above	https://www.ncbi.nlm.nih.gov/bioproject/PRJNA728470		https://www.atcc.org/products/all/MSA-1003.aspx

Supplementary Table 1: Linked-read datasets of mock microbiome communities used demonstrate the utility of read cloud/barcode deconvolution prior to de novo assembly.

Dataset	Num. Barcoded Reads	Search Distances	Num. Reads Deconvolved	Prop. Reads Deconvolved	Avg. Cloud Purity	Std. Dev.	Avg. Cloud Entropy	Std. Dev.	Avg. Cloud Size	Std. Dev.	Under	Complete	Over
MOCK5 10x	91,101,472	No_Deconv	NA	NA	0.53	0.16	1.61	0.52	63.51	51.64	0.98	0.02	0
MOCK5 10x	91,101,472	Fragments	69,702,820	0.7651119	0.99	0.05	0.03	0.14	18.47	19.26	0.06	0.91	0.03
MOCK5 10x	91,101,472	5000	46,136,720	0.50643221	0.89	0.22	0.37	0.7	12.18	15.26	0.25	0.04	0.71
MOCK5 10x	91,101,472	10000	60,297,800	0.66187515	0.87	0.23	0.42	0.71	12.76	15.77	0.29	0.06	0.65
MOCK5 10x	91,101,472	15000	67,592,474	0.74194711	0.85	0.23	0.47	0.72	14.11	19.21	0.34	0.08	0.58
MOCK5 10x	91,101,472	20000	81,388,754	0.89338572	0.83	0.24	0.51	0.72	15.44	22.29	0.38	0.1	0.52
MOCK5 LoopSeq	75,107,814	No_Deconv	NA	NA	0.93	0.11	0.31	0.31	269.18	356.67	0.68	0.32	0
MOCK5 LoopSeq	75,107,814	Fragments	68,345,024	0.75020768	0.98	0.07	0.06	0.21	116.19	252.59	0.14	0.82	0.04
MOCK5 LoopSeq	75,107,814	5000	68,658,974	0.91413889	0.88	0.21	0.4	0.7	101.44	225.71	0.35	0.14	0.51
MOCK5 LoopSeq	75,107,814	10000	68,884,270	0.91713853	0.87	0.21	0.42	0.69	103.16	229.15	0.39	0.15	0.47
MOCK5 LoopSeq	75,107,814	15000	69,051,634	0.91936685	0.87	0.21	0.44	0.68	104.95	232.22	0.41	0.15	0.44
MOCK5 LoopSeq	75,107,814	20000	68,345,024	0.75020768	0.86	0.21	0.45	0.67	106.42	234.67	0.43	0.15	0.42
MOCK20 10x	94,151,528	No_Deconv	NA	NA	0.38	0.15	2.28	0.62	186.49	129.32	0.97	0.03	0

MOCK20 10x	94,151,528	Fragments	73,646,746	0.78221509	0.99	0.06	0.05	0.18	29.31	29.26	0.13	0.83	0.04
MOCK20 10x	94,151,528	5000	67,862,032	0.72077462	0.94	0.18	0.22	0.64	13.56	22.69	0.13	0.05	0.83
MOCK20 10x	94,151,528	10000	77,573,094	0.82391753	0.91	0.21	0.32	0.73	17.24	31.17	0.19	0.06	0.76
MOCK20 10x	94,151,528	15000	81,429,216	0.86487408	0.89	0.23	0.39	0.79	20.32	37.96	0.22	0.07	0.71
MOCK20 10x	94,151,528	20000	83,535,484	0.88724512	0.87	0.24	0.43	0.82	22.73	43.16	0.25	0.08	0.68
MOCK20 TELLSeq	100,000,000	No_Deconv	NA	NA	0.39	0.15	2.37	0.59	160.45	110.51	0.98	0.02	0
MOCK20 TELLSeq	100,000,000	Fragments	78,420,964	0.78420964	1	0.02	0	0.05	23.83	27.65	0.01	0.99	0
MOCK20 TELLSeq	100,000,000	1000	28,365,704	0.28365704	0.88	0.24	0.44	0.9	24.53	48.33	0.22	0.02	0.76
MOCK20 TELLSeq	100,000,000	2000	39,754,436	0.39754436	0.89	0.23	0.41	0.86	21.68	38.18	0.22	0.03	0.76
MOCK20 TELLSeq	100,000,000	4000	52,929,128	0.52929128	0.88	0.24	0.44	0.87	21.42	31.47	0.24	0.04	0.72
MOCK20 TELLSeq	100,000,000	5000	57,021,308	0.57021308	0.87	0.24	0.47	0.89	21.96	30.74	0.25	0.04	0.71

Supplementary Table 2: Extended version of Table 1 from the main text with additional search distances for Ariadne deconvolution. The 'Std. Dev' columns correspond to the parameter immediately to the left. The 'Under, Complete, and Over' columns refer to the proportion of total original or deconvolved read clouds that were over- or under-deconvolved, or completely and exactly comprised of all of the reads from a single inferred genomic fragment.

Dataset	Species	Deconv. Method	Outlier Largest Alignment
MOCK20 10x	Escherichia coli	Reference	2,410,209
MOCK20 10x	Pseudomonas aeruginosa	Reference	6,234,231
Dataset	Species	Deconv. Method	Outlier NA50
MOCK20 10x	Pseudomonas aeruginosa	Reference	6,234,231
MOCK20 10x	Streptococcus agalactiae	Reference	2,049,091
MOCK20 TELL-Seq	Bacillus cereus	Reference	1,783,115
MOCK20 TELL-Seq	Staphylococcus epidermidis	Reference	1,503,747
MOCK20 TELL-Seq	Streptococcus agalactiae	Reference	2,047,925
MOCK20 TELL-Seq	Streptococcus mutans	Reference	2,012,488

Supplementary Table 3: Extension of Table 2 in the main text. Outlier values of the relative largest alignment and relative rate of misassembled bases not represented in Figure 2 in the main text. Largest alignment is the largest alignment to the reference sequences from scaffolds obtained from the deconvolution method. The larger the relative alignment, the longer it is relative to the no-deconvolution scaffolds.

Dataset	Max. Frag. Len. (kbp)	Avg. Purity	Avg. Entropy	Avg. Size	Num. Clouds
MOCK5 LoopSeq	100	0.96 ± 0.10	0.08 ± 0.34	114.72 ± 300.56	638,992
MOCK5 LoopSeq	200	0.98 ± 0.07	0.06 ± 0.21	116.19 ± 252.59	637,012
MOCK5 LoopSeq	400	0.99 ± 0.06	0.06 ± 0.08	120.54 ± 276.18	636,983
MOCK20 TELL-Seq	100	0.99 ± 0.04	0.01 ± 0.07	23.85 ± 32.88	4,069,335
MOCK20 TELL-Seq	200	1 ± 0.02	0 ± 0.05	23.83 ± 27.65	4,067,621
MOCK20 TELL-Seq	400	0.99 ± 0.04	0.01 ± 0.02	25.83 ± 20.62	4,066,890

Supplementary Table 4: Halving and doubling the maximum fragment length does not meaningfully change the quality of reference-deconvolved read clouds.

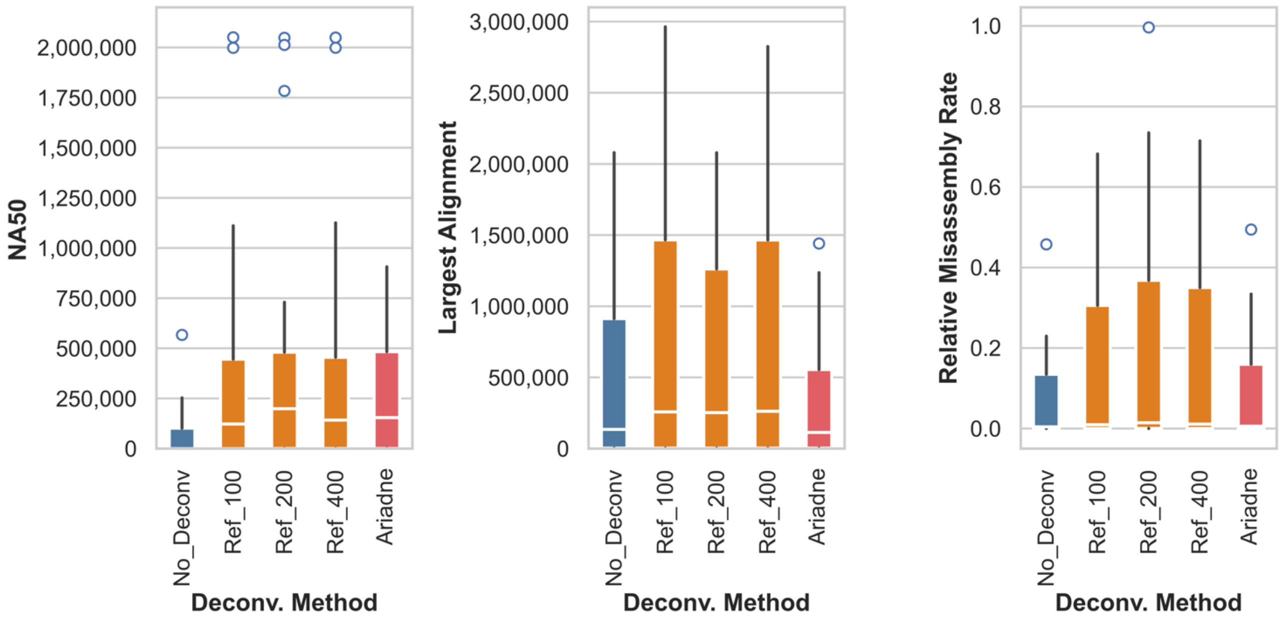

Supplementary Figure 2: Halving and doubling the maximum fragment length does not meaningfully change the quality of de novo assembly using reference-deconvolved linked-reads. Shown here are the NA50, largest alignments, and relative misassembly rate of the MOCK20 TELL-Seq reference-deconvolved assembly.

Dataset	Deconv. Method	Avg. Cloud Purity	Std. Dev.	Avg. Cloud Entropy	Std. Dev.	Avg. Cloud Size	Std. Dev.	Num. Clouds
20-species	None	0.24	0.08	2.77	0.44	58.13	18.61	1 720 220
20-species	Reference	1	0.01	0	0.02	6.08	1.24	16 038 712
20-species	Ariadne	0.87	0.26	0.43	0.89	8.32	9.35	12 025 136
50-species	None	0.29	0.15	2.39	0.66	30.37	15.35	3 290 686
50-species	Reference	1	0.0029	0.0001	0.01	5.86	1.77	15 095 869
50-species	Ariadne	0.45	0.32	1.85	1.14	23.57	16.81	4 237 573
100-species	None	0.27	0.15	2.44	0.69	29.61	15.34	3 374 189
100-species	Reference	1	0.001	0	0.0059	5.63	1.38	15 427 457
100-species	Ariadne	0.35	0.26	2.19	0.97	26.55	16.24	3 762 776

Supplementary Table 5: Read cloud summary statistics for the LRSim-simulated 10x datasets of 20, 50 and 100 species. For Ariadne deconvolution, we used a search distance of 5 kbp and for reference-based deconvolution we used 200 kbp as the maximum fragment length.

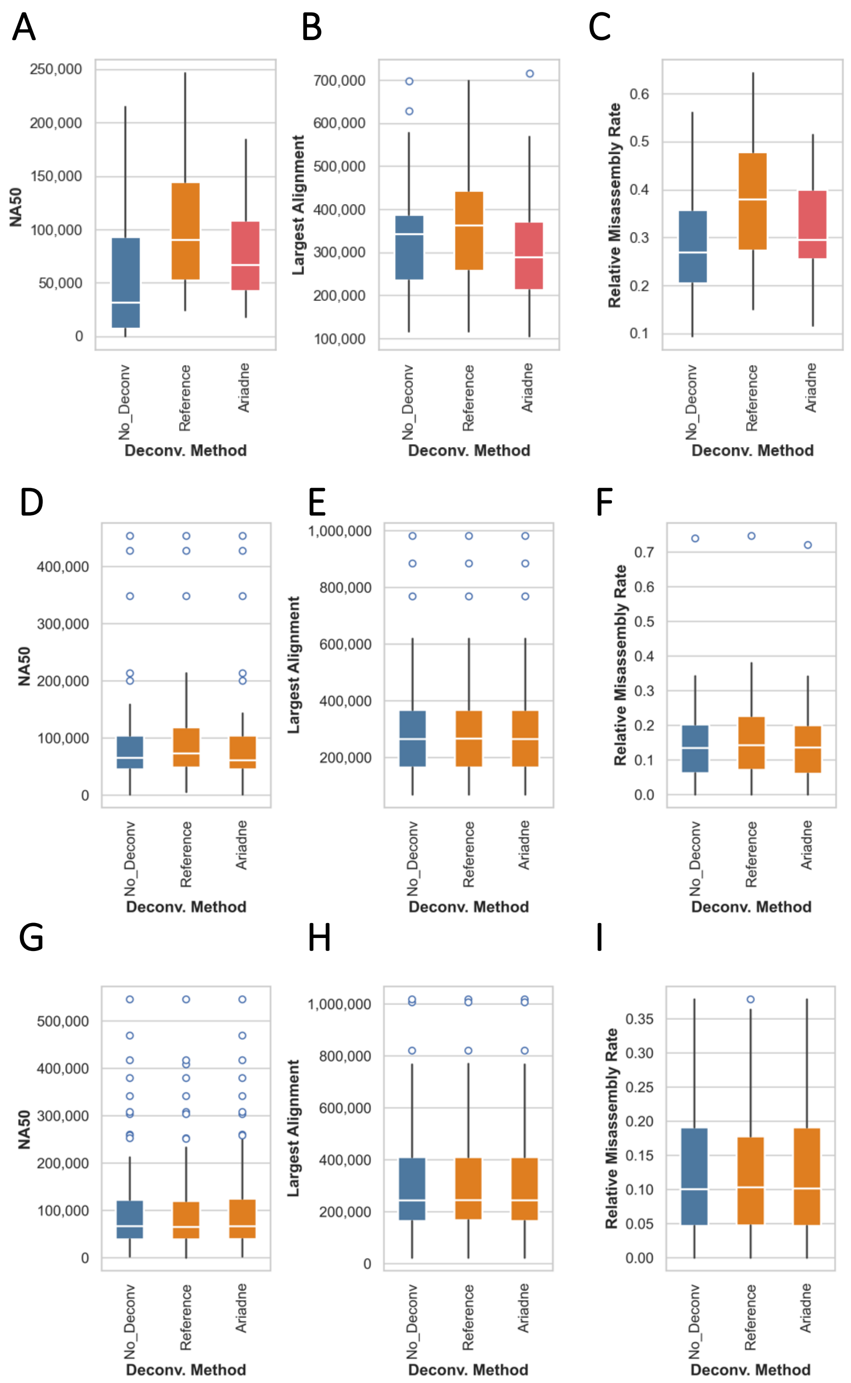

Supplementary Figure 3: While the 20-species simulated 10x dataset results **(A, B, C)** are similar to those from the real 20-species dataset (Figure 2 column 3) There is not much of a difference between metagenomic assembly with or without deconvolution, reference or otherwise, with linked-read datasets simulated from **(D, E, F)** 50 or **(G, H, I)** 100 species.

Promotion	No Deconv.	Reference	Prop. Improvement	Ariadne	Prop. Improvement
R->D	330,720	87,665	-0.74	164,568	-0.50
R->P	207	380	0.84	1,236	4.97
R->C	87	633	6.28	670	6.70
R->O	17	262	14.41	142	7.35
R->F	180	155,061	860.45	123,607	**685.71**
R->G	57	68,966	1208.93	52,394	**918.19**
R->S	176	67,165	380.62	34,315	**193.97**
D->P	845	2,201	1.61	8,371	8.91
D->C	474	3,236	5.83	3,421	6.22
D->O	86	2,687	30.24	1,140	**12.26**
D->F	574	285,153	495.78	171,430	**297.66**
D->G	306	95,279	310.37	45,947	**149.15**
D->S	854	683,635	799.51	115,444	**134.18**
P->C	14,402	4,198	-0.71	15,509	0.08
P->O	504	591	0.17	1,236	1.45
P->F	5,979	64,429	9.78	51,688	7.65
P->G	893	38,593	42.22	22,933	**24.68**
P->S	11,542	106,922	8.26	71,363	5.18
C->O	2,106	889	-0.58	2,202	0.05
C->F	67,336	104,792	0.56	99,832	0.48
C->G	14,669	35,311	1.41	28,343	0.93
C->S	177,362	239,726	0.35	217,352	0.23
O->F	390,219	390,255	0.00	390,090	0.00
O->G	88,250	91,886	0.04	90,476	0.03
O->S	612,701	651,838	0.06	645,002	0.05
F->G	1,765,745	1,763,894	0.00	1,763,052	0.00
F->S	618,889	621,663	0.00	622,575	0.01
G->S	6,167,224	6,196,310	0.01	6,190,811	0.00

Supplementary Table 6: Read cloud deconvolution specifically promotes reads to low taxonomic ranks such as genus and species in the MOCK20 TELL-Seq dataset. The column 'Promotion' indicates the promotion of a paired read *i* from initial rank X to rank Y as 'X->Y' using deconvolved read cloud information. The abbreviations are as follows: Root (R), kingdom/domain (D), phylum (P), class (C), order (O), family (F), genus (G), species (S). The columns 'Prop. Improvement' are calculated by taking the difference between the number of reads promoted using the enhanced read clouds and the number of reads promoted using the original read clouds, divided by the latter. Taxon promotions where the proportion of promoted reads in the Ariadne vs. non-deconvolved datasets is greater than 10 are bolded.

Initial Taxon	Promoted Taxon	No Deconv.	Ariadne	Num. Reads Difference
Root	Bacteria	218,728	48,874	-169,854
Root	Proteobacteria	17,279	29,216	11,937
Root	Alphaproteobacteria	5	5	0
Root	Rhodobacterales	1	1	0
Root	Rhodobacteraceae	7	12	5
Root	Rhodobacter	5	285	280
Root	Rhodobacter sphaeroides	492	3,587	3,095
Bacteria	Proteobacteria	43,404	62,234	18,830
Bacteria	Alphaproteobacteria	35	42	7
Bacteria	Rhodobacterales	29	84	55
Bacteria	Rhodobacteraceae	71	268	197
Bacteria	Rhodobacter	64	219	155
Bacteria	Rhodobacter sphaeroides	3,925	58,845	54,920
Proteobacteria	Alphaproteobacteria	51	219	168
Proteobacteria	Rhodobacterales	40	176	136
Proteobacteria	Rhodobacteraceae	128	525	397
Proteobacteria	Rhodobacter	88	390	302
Proteobacteria	Rhodobacter sphaeroides	9,041	101,959	92,918
Alphaproteobacteria	Rhodobacterales	1,232	486	-746
Alphaproteobacteria	Rhodobacteraceae	2,529	994	-1,535
Alphaproteobacteria	Rhodobacter	3,652	928	-2,724
Alphaproteobacteria	Rhodobacter sphaeroides	354,827	360,909	6,082
Rhodobacterales	Rhodobacteraceae	2,783	1,026	-1,757
Rhodobacterales	Rhodobacter	3,162	817	-2,345
Rhodobacterales	Rhodobacter sphaeroides	362,506	368,000	5,494
Rhodobacteraceae	Rhodobacter	6,679	1,679	-5,000
Rhodobacteraceae	Rhodobacter sphaeroides	751,157	758,823	7,666
Rhodobacter	Rhodobacter sphaeroides	694,571	699,793	5,222

Supplementary Table 7: A case study of Rhodobacter (Cereibacter, using Kraken2's database) sphaeroides taxonomic promotion. Every taxon name under 'Promoted Rank' is a taxon *R. sphaeroides* is classified under. The column 'Num. Reads Difference' is the difference between the number of reads promoted using the enhanced read clouds and the number of reads promoted using the original read clouds.

		EMA		Minerva		Reference	
Dataset	Num. Barcoded Reads	Num. Reads Deconvolved	Prop. Reads Deconvolved	Num. Reads Deconvolved	Prop. Reads Deconvolved	Num. Reads Deconvolved	Prop. Reads Deconvolved
MOCK5 10x	91,101,472	48,191	0.00049431	Timed out after 3 days.	NA	69,702,820	0.7651119
MOCK5 LoopSeq	75,107,814	Invalid barcode AAA...AA whitelisted---exiting!	NA	121,238	0.00161419	54,869,732	0.73054625
MOCK20 10x	94,151,528	199,488	0.00199488	129,832	0.00137897	73,646,746	0.78221509
MOCK20 TELLSeq	100,000,000	13,553	0.00013553	Timed out after 3 days.	NA	78,420,964	0.78420964

Supplementary Table 8: Neither EMA nor Minerva deconvolved an appreciable part of the linked-read datasets. As such, they were omitted from the main text analysis. The specific reasons for runs that did not generate any deconvolved reads are listed in the table.

Similar to the full 97-million read dataset, there is at least a 6.2-fold increase in the number of single-origin read clouds after Ariadne deconvolution relative to no deconvolution, regardless of the search distance. (Figure 2 top vs. bottom left). In comparison, Minerva increases the number of single-origin read clouds by 81%. Minerva is capable of deconvolving read clouds with sufficient k-mer dissimilarities between reads that originated from different fragments. However, only 4% of reads satisfies this criterion (Figures 2 bottom center vs. bottom right). This is also demonstrated by the minimal gain in average purity by the set of all read clouds that Minerva was applied to, in comparison to the subset that it was able to deconvolve (Supplementary Figure 3 and Table 6, 'Minerva' vs. 'With Filter' respectively). Thus, most of the original read clouds remain un-deconvolved with the application of Minerva. While Minerva took 15.5 hours and 163 GB of RAM, Ariadne consumed, at a maximum (search distance of 35 kbp), 4 hours and 62 GB of RAM on 20 CPUs.

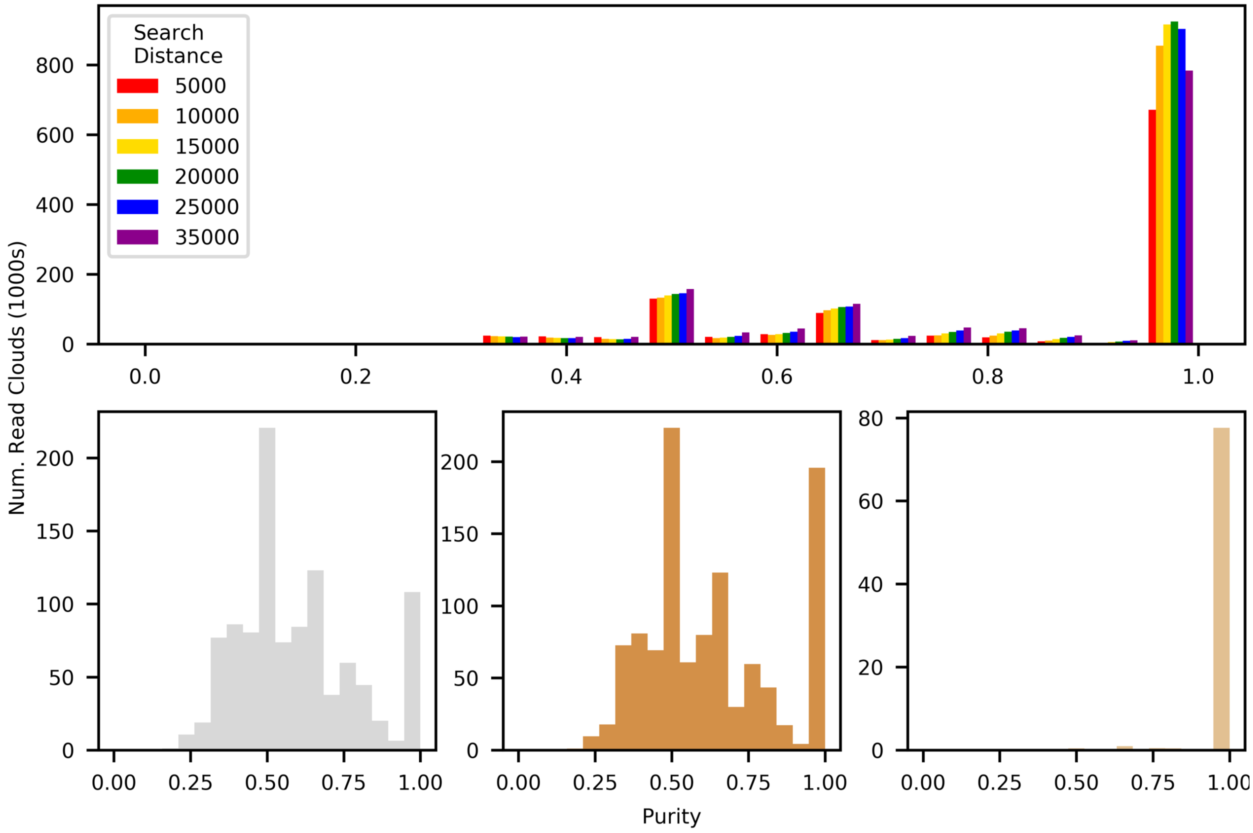

Supplementary Figure 4: Performance comparison between Ariadne and Minerva on a 20-million read subset of the MOCK5 10x dataset. Top: Purity of Ariadne-deconvolved enhanced read clouds. Bottom left: Purity of the original read clouds. Bottom center: Purity of the entire set of read clouds after applying Minerva. This set includes read clouds that Minerva was unable to deconvolve as well as the read clouds Minerva was able to act upon. Bottom right: Purity of Minerva-deconvolved read clouds only.

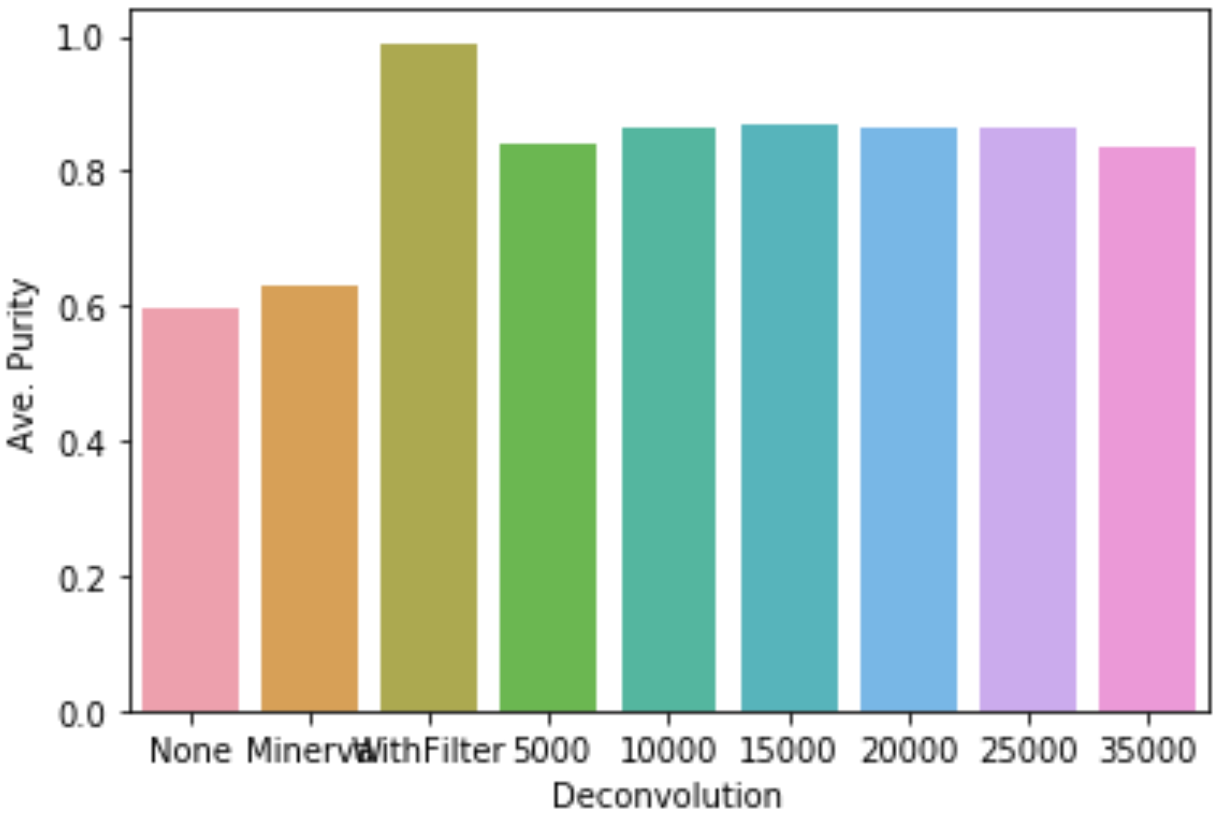

Supplementary Figure 5: Average purity of non-deconvolved, Minerva-deconvolved with non-deconvolved, Minerva-deconvolved only (i.e.: filtered out all read clouds that could not be deconvolved by Minerva), and Ariadne-deconvolved read clouds. Ariadne deconvolution was carried out with multiple search distances (5 - 35 kbp). The same 20-million read subset of MOCK5 10x as above was used.

No deconv.	Minerva	With Filter	5000	10000	15000	20000	25000	35000
0.5962	0.6307	0.9900	0.8389	0.8652	0.8686	0.8494	0.8433	0.8363

Supplementary Table 9: Average purity of non-deconvolved, Minerva-deconvolved with non-deconvolved, Minerva-deconvolved only, and Ariadne-deconvolved read clouds. The same 20-million read subset of MOCK5 10x as above was used.
