# Supplementary material for: Ariadne: synthetic long read deconvolution using assembly graphs
Source: Genome Biol. 2023 Aug 28;24:197. doi: 10.1186/s13059-023-03033-5 (PMC10463629; doi:10.1186/s13059-023-03033-5)
Supplement: Supplementary file 2 — Additional file 2. The review history. [file 13059_2023_3033_MOESM2_ESM.docx]

**1^st^ round**

**Reviewer 1**

SUMMARY

The authors present a synthetic long read (SLR) deconvoluting algorithm with its implementation, Ariadne. The objective of Ariadne is to separate (deconvolute) reads tagged with the same UMI but coming from different fragments. Such deconvolution can help downstream algorithms use such data to produce better genome assemblies or structural variation calling.

Ariadne is implemented as a module in cloudSPAdes and uses the de bruijn graph (DBG) representation of cloudSPAdes as the underlying data structure to perform the deconvolution. Reads with the same UMI that appear close in the graph are inferred to be from the same read cloud. Here, the graph distance is inferred from the sum of edge lengths in the graph, where edges hold strings formed from collapsed non-branching paths in the DBG.

The authors show that Ariadne can deconvolute the majority of reads in three out of four datasets (exception being the LoopSeq dataset). The authors further show that this convolution, to some extent, improves the genome assemblies of the datasets. In addition, two of the four datasets are generated by the authors and could be useful to the community working on SLR data within metagenomics. These are welcomed contributions.

The two most substantial concerns I have about the study are the impact of deconvolution in genome assembly and the resource requirements of the tool. These concerns are provided below in comments 1 and 2 — a list of additional comments further below.

MAJOR

1. SLR deconvolution is not the final step in any analysis. As such, the main motivation for performing deconvolution is to help a downstream application such as genome assembly, structural variant detection, or other application. Therefore, the impact of SLR deconvolution as an algorithmic problem is mostly driven by downstream results. The authors have chosen metagenomic assembly as the application. However, the final assembly statistics seems to be similar to non-deconvoluted read clouds (Figure 2; similar NA50 and misassembly rate). This limits the impact of performing deconvolution. If there is a 'significant improvement' (non-statistical meaning) as the authors claim, it is not well illustrated in Figure 2. There may be other bioinformatic applications where the Ariadne-enhanced clouds demonstrate a significant improvement. For example, their predecessor, Minerva, presented improved downstream results for "specificity of taxonomic assignments and of k-mer-based clustering."

2. The runtime is substantial. It can take several days to complete a deconvolution (Suppl Table 3). In comparison, the cloudSPAdes assembly only takes a small fraction of Ariadne's runtime. Memory usage is also high in some instances. In combination with the modest assembly improvement, this could limit the tool's use.


OTHER SIGNIFICANT REMARKS


3. In section 5.4, the authors reason around the probability that two different fragments with the same UMI are unlikely to overlap using a model from [23]. They also claim that the only drawback with their model is that it does not model repeats. Some concerns:
- If different strains from the same species are considered different contigs to be assembled separately, I assume that repeats in the DBG (from conserved regions) should not be uncommon. This would make it much more likely that two fragments are within search distance d of each other in the cloudSPAdes graph. Therefore, the accuracy of the model seems questionable.
- It seems that the authors also evaluate the accuracy of this model to some extent in Suppl. Table 1 (cloud purity). Table1 indicates that with a larger distance, more fragments overlap in the graph. This seems to go against the claim that "random fragments ... are unlikely to be similar in terms of sequence to overlap on the assembly graph". Also, if this claim was true, why would SLR deconvolution benefit the assembler, as any two fragments with same UMI would be distant in the graph and could instead be easily separated during the assembly process?
- "For metagenomic datasets, at least 95% of read clouds consist of fragments that do not overlap in this model." Which analysis is this claim coming from?


4. For the analysis of entropy (H) and purity (P), it would be relevant to consider a purely simulated dataset. Bowtie2 will be subject to alignment misplacements and Ariadne to deconvolution errors. This could also answer whether the authors' model (from remark 3) holds for these types of datasets.

5. No explicit definition of a "misassembly rate" metric is given in the text.

6. NA50 metric does not consider the assembly length - why is NGA50 metric not used instead? Also, in table 3, I am missing the reference genome sizes for the organisms to deduce whether the numbers are substantial. And why are only some genomes out of the 20 included?

7. It would be informative to include SLR deconvolution results on more complex metagenomic datasets than MOCK20, as there are publicly available datasets e.g. human gut sample.

8. The authors show that the reference deconvolution procedure results in a significantly higher misassembly rate. Could this be alleviated by changing the maximum fragment length parameter currently set at 200 kbp, thus obtaining a more accurate gold standard deconvolution?


MINOR

Methods

1. Vertex v_j is not defined in the equation describing the forward Dijkstra graph on page 9
2. Which dataset is used for Supplementary Figure 3?
3. Page 10, Step2B: "This process is conducted via binary search of sorted vertex names in the forward and reverse Dijkstra graphs," Sorted with respect to distance from start node? Overall, this procedure could be described in more detail.
4. Page 10, Step2B: "if one of the following conditions holds" and "Else, if neither of the above is true," I only see one condition in the paragraph.
5. Page 10, Step2B: Is it correct that only an upstream read (w.r.t. the assembly graph) can identify downstream reads as part of the same fragment? That is, if the true genomic order of reads in a read cloud is r_1, r_2, r_3, then r_1 can identify both r_2 and r_3 as belonging to the same cloud, but r_2 can only identify r_3. Thus, if the algorithm starts with r_2 and identifies r_3 as belonging to the same fragment, when exploring r_1 as r_i, one only need to connect r_1 with r_2. More details around this could be given.
6. Page 10, section 5.2.3: There is no motivation given for described processing of enhanced clouds composed of a single pair of reads. Nothing indicates that adding such clouds to the smallest enhanced read cloud with the same UMI would result in a set of reads originating from the same fragment if more than one non-trivial enhanced read cloud with the same UMI is present.
7. Page 11, Section 5.4: "We provide a short summary of the mathematical model that justifies the modelling" In the text that follows, no mathematical model is given. Either change to "[23] provided a mathematical model..." Or describe the model.

Results

8. "We termed this method 'reference deconvolution' as it represents the maximum likelihood inference of the genomic fragments" It is unclear what model the 'maximum likelihood inference' is referring to, and I believe in such a case, it would rather be 'maximum likelihood estimate'. Either a model should be provided, or it is easier to avoid the term' maximum likelihood' here.
9. Table 4. Clarify the meaning of the asterisk, particularly "no associated NA50 ".
10. Section 2.3: "Furthermore, reference-based deconvolution generates a similar number of clouds of a similar size to Ariadne". The MOCK20 10X number of clouds (3.1M vs 6.9M) stands out significantly from this claim and should be discussed or at least mentioned.
11. Section 2.3: "The ideal deconvolution based on reference-mapped positions is shown in the Figure 1 middle row, where there are extremely few clouds with P < 1." This cannot be inferred from the dot-plot figure. For example, from MOCK20 there are many dots below 1, although I am guessing the density of dots at 1 is much higher.


TEXT

- Typo "SLR datsets" on page 7
- "Additionally, is an extension of the EMA approach that makes"
- "Chimeric contigs are hazardous for downstream analyses, since as the binning of contigs into metagenomic-assembled genomes"
- "...we will be able to all reads with the same UMI..."
- "within the maximum search distance d the 3'-terminal"

**Reviewer 2**

In this paper, Mak et al. explore deconvolution of synthetic long read/linked read data sets (for maintaining haplotype/genome linkages across long distances) using assembly graphs.

From reading the paper, I think the authors have implemented a high quality approach that yields good results! The technical writing is good quality, and the implementation seems great! Overall the approach makes sense and provides much improved results.

However, I think the paper needs to more clearly state what it is doing up front. I was initially quite confused when I was reading the paper because I had thought ARIADNE was about metagenomic deconvolution, and this is not mentioned in the title, the abstract, or the background. Then I read further and realized it _was_ about metagenomic deconvolution, but this hadn't been mentioned clearly until fairly far into the text! Likewise the conclusion doesn't mention microbiomes or metagenomes until the second sentence.

So my main suggestion is that the title, abstract, background, discussion, and conclusion clearly identify the problem using terms like "microbiome" and "metagenome", not just "complex genomic mixtures" and "complex samples", and do so up front.

Similarly, the first paragraph of the Discussion is a puzzling disconnect from the paper. I would suggest making a general statement first that sums up the results for readers who jump to this part of the text to understand what the paper is trying to say.

A few other comments -

* p1 line ~48 second column, "When sequencing depth is low, the assembly graph contains many ambiguous junctions comprised of sequence material from multiple genetically similar species and/or strains." This is clearly true of high depth sequencing too - presumably the authors mean it's harder to deconvolute at low depths? Or something else? Please clarify.

* p2 line 32 colum 2, For "Many taxonomic lineages identified ... are not represented in reference databases", I was surprised that Hug et al. was the only reference here. Maybe one or another of the large scale studies (like the various Almeida papers) would be good to add here, if there is room in the citations.

* The second half of that paragraph is also poorly motivated, I think - reference based analyses will not _bias_ towards the reference, they will _completely ignore_ anything not in the reference. I am not sure exactly what the point of this sentence is? I feel like there are more straightforward ways to make the case for de novo assembly.

* p3 line 10 second column, a reference or word is missing after "Additionally."

* p3 line 33 second column - traditionally de Bruijn assembly graphs (presumably compact DBGs?) do not contain linkage information. Although the ones produced by cloudSPADes might? Please state if so.

* p5 line 57 column 1 - "significantly positive differences" could be rephrased - do you mean "positive differnces that were statistically significant" or "large differences that were positive"?

**Authors’ response**

Reviewer #1:

1. SLR deconvolution is not the final step in any analysis. As such, the main motivation for

performing deconvolution is to help a downstream application such as genome assembly,

structural variant detection, or other application. Therefore, the impact of SLR deconvolution as

an algorithmic problem is mostly driven by downstream results. The authors have chosen

metagenomic assembly as the application. However, the final assembly statistics seems to be

similar to non-deconvoluted read clouds (Figure 2; similar NA50 and misassembly rate). This

limits the impact of performing deconvolution. If there is a 'significant improvement' (non-

statistical meaning) as the authors claim, it is not well illustrated in Figure 2. There may be other

bioinformatic applications where the Ariadne-enhanced clouds demonstrate a significant

improvement. For example, their predecessor, Minerva, presented improved downstream

results for "specificity of taxonomic assignments and of k-mer-based clustering."

As the reviewer suggested we used taxonomic assignments as an additional downstream

application. We have added a full section about using enhanced read clouds to improve short-

read taxonomic classification in the manuscript (section 2.8). We found that the promotion of

classifications from high ranks such as kingdom and phylum to extremely specific ranks (of

species that are known to be in the original sample's compositions) was substantially improved

with additional deconvolution. Please see page 7 for more information.

2. The runtime is substantial. It can take several days to complete a deconvolution (Suppl Table

3). In comparison, the cloudSPAdes assembly only takes a small fraction of Ariadne's runtime.

Memory usage is also high in some instances. In combination with the modest assembly

improvement, this could limit the tool's use.

This really bothered us as well. In the revised version, we were able to cut the maximal runtime

from a few days to only a few hours, which is a huge speedup. In the end, LM refactored the

entire algorithm and it performs significantly better now. There were three pain points in the

initial implementation:

1) While the implementation was The cloud-solving step was inefficient. For each read cloud

containing r reads that are mappable to the assembly graph. Each read r has a set of vertices

that it is within the search distance of i.e.: 'neighboring' vertices. To find maximally connected

subsets within the read cloud, we have to make r(r - 1) comparisons between the each read's set

of neighborhood vertices. Initially, every time each read r_i and r_j were considered, the

assembly graph was Djikstra-searched for r_j's neighborhood vertices, which is at worst r(r - 1)/2

Djikstra searches, most of which are repetitive.

2) Though the cloud-solving step was multi-threaded, the FastQ-writing step was single-threaded

to prevent race conditions. As previously implemented, the writing step was not minimalist (i.e.:

contained string-formatting code as well as actually writing to the FastQ), which prevented

threads from being released back to the pool efficiently.

3) Ariadne required the rest of the cloudSPAdes infrastructure to directly provide it with an

assembly graph, and had to be run as a part of cloudSPAdes.

By addressing these three issues, we were able to cut the maximal runtime from a few days to a

few hours:

1) Instead of looking up read r_j's neighboring vertices every time it's compared to a read r_i, I

simply saved them in a list beforehand, and treated each of the reads as a putative enhanced

cloud initially. I then merged reads (or sets of reads) any time I found overlaps between

neighborhood vertex sets, and deleted individual reads as they were merged into sets (or kept

them as-is if they didn't neighbor any other reads).

2) I minimized the single-threaded writing step and moved formatting to the multi-threaded

cloud-solving step. The only single-threaded code remaining is flushing the buffer.

3) As part of the refactoring process, we can now run Ariadne completely separately from

cloudSPAdes, with or without an existing assembly graph. If an assembly graph is available, that

step can be skipped too.

We uploaded the new software to the project GitHub page. We did not change the core

algorithms in the manuscript but these new improvements were implemented and documented

in the new release of our software on GitHub.

Please see Table 7 on page 21 for a breakdown of the new computational resource usages.

3. In section 5.4, the authors reason around the probability that two different fragments with the

same UMI are unlikely to overlap using a model from [23]. They also claim that the only

drawback with their model is that it does not model repeats. Some concerns:

- If different strains from the same species are considered different contigs to be assembled

separately, I assume that repeats in the DBG (from conserved regions) should not be uncommon.

This would make it much more likely that two fragments are within search distance d of each

other in the cloudSPAdes graph. Therefore, the accuracy of the model seems questionable.

If strain inference were the goal, then de novo assembly is probably not the step to start or end

with, depending on the nature of the sample. If there is significant intra-species genetic variation

(relative to a known reference genome) and sufficient sequencing depth to capture it, some

form of linked-read mapping to the known reference genome, followed by variant-calling and

some single-species strain/haplotype inference method is probably the most reliable way to

profile the presence of multiple strains. If there is suspected sub-species variation but either no

known reference genome or (likely) insufficient sequencing depth to fully disambiguate each

strain, then de novo assembly will likely compress intra-species heterogeneity to a single

sequence representation (i.e.: contig) that will need to be recovered later on through read

mapping as above. For example, there is a tool called CMSeq

(https://github.com/SegataLab/cmseq) specifically designed for a first-pass summary of strain

heterogeneity within MAGs (sets of contigs that are predicted to come from a single species). If

the sequencing coverage is low and the support for individual edges in an assembly graph fork is

not high, de novo assembly with or without linked reads is probably not going to recover strains.

In our opinion, the barcode model isn't applicable to strain inference with de novo assembly as a

starting point.

- It seems that the authors also evaluate the accuracy of this model to some extent in Suppl.

Table 1 (cloud purity). Table1 indicates that with a larger distance, more fragments overlap in the

graph. This seems to go against the claim that "random fragments ... are unlikely to be similar in

terms of sequence to overlap on the assembly graph".

Our working hypothesis is that reads from the same assembly graph neighborhood theoretically

originate from the same genomic fragment. The results- increased search distance creating

lower purity clouds i.e.: pulling in reads that do not originate from the same species- seem to

disagree with that premise. We have two potential explanations for this:

1) There is only a very slight drop in purity when increasing search distance that can be

explained by the inclusion of reads that do not map to any reference genome due to

strain variation.

a. Note that reference-deconvolved clouds are not perfectly pure either due to

these unmapped reads (in the case that one of the reads in a pair maps and the

other one does not).

2) Larger search distances may have modeled a larger genomic fragment than was actually

created during the wet lab fragmentation process.

(Assuming that the tables referred to are Table 3 in the main text and Supp. Table 2).

Also, if this claim was true, why would SLR deconvolution benefit the assembler, as any two

fragments with same UMI would be distant in the graph and could instead be easily separated

during the assembly process?

As mentioned previously in this response, 3' UMI/barcode information helps cloudSPAdes

disambiguate paths through the assembly graph (i.e.: contigs). However, unlike Ariadne, the

path-finding algorithm is not bounded by a search distance, and each subsequent edge that is

added to the existing path is added based on the similarity between the set of barcodes that the

edge is associated with vs. that of the edge. Thus, erroneous edges can be added to the path if

the barcode sets pass a similarity threshold. However, if for example fragments m and n both

original carried the same barcode but were located at distance greater than the search distance,

assigning reads (mapping to fragment m a different barcode than n would by extension give the

edges associated with fragment m and n a different barcode, preventing them from being joined

without sufficient evidence from other barcodes.

- "For metagenomic datasets, at least 95% of read clouds consist of fragments that do not

overlap in this model." Which analysis is this claim coming from?

Our apologies! This is a typo and the actual number from Danko et al. 2019 is 99% (page 12),

from the equation that defines the probability that two fragments Fw,x, Fy,z are the same given

that their genomes Gw, Gy are the same. This was corrected in the text and we added the

citation.

4. For the analysis of entropy (H) and purity (P), it would be relevant to consider a purely

simulated dataset. Bowtie2 will be subject to alignment misplacements and Ariadne to

deconvolution errors. This could also answer whether the authors' model (from remark 3) holds

for these types of datasets.

We have added a full section about simulating higher-complexity linked-read datasets (section

2.7 starting on page 6) with 50 and 100 species, and found that while Ariadne deconvolution

does improve read cloud purity, the improvements were not as distinct as with the mock

metagenome datasets with fewer species, indicating a possible upper bound of performance as

to search distance-based deconvolution in terms of being able to specifically cluster sequences

to species-specific clouds.

5. No explicit definition of a "misassembly rate" metric is given in the text.

The relative misassembly rate was defined on page 5 on the bottom left side as ‘the number of

misassembled bases divided by the total assembly length’.

6. NA50 metric does not consider the assembly length - why is NGA50 metric not used instead?

Also, in table 3, I am missing the reference genome sizes for the organisms to deduce whether

the numbers are substantial. And why are only some genomes out of the 20 included?

We went back and checked the NA50 vs. NGA50 MetaQUAST tables while the assembly size-

scaled NGA50 values were different than the NA50 ones, there was not much of a difference in

trends (ex. reference-based deconvolution generally outperforms Ariadne-based deconvolution

which generally outperforms no deconvolution). See below for a tabular comparison of the

MOCK5 LoopSeq dataset NA50 vs. NGA50 values.

Assemblies No_Deconv Reference_100 Reference_200 Reference_400 Ariadne

Escherichia_coli_str._K_12 238086 348213 283465 427376 422567

Porphyromonas_gingivalis_ATCC_33277 41811 139573 133740 138089 100399

Rhodobacter_sphaeroides_ATCC_17029 363460 834700 834700 834709 455792

Streptococcus_mutans_UA159 16457 37396 37396 37396 14585

Assemblies No_Deconv Reference_100 Reference_200 Reference_400 Ariadne

Escherichia_coli_str._K_12 238086 427376 343795 427376 422567

Porphyromonas_gingivalis_ATCC_33277 88637 139573 122010 138089

122112

Pseudomonas_aeruginosa_strain_SP4527 - - - - -

Rhodobacter_sphaeroides_ATCC_17029 378391 834700 834700 834709

728814

Streptococcus_mutans_UA159 27710 29436 29436 29436 26792

Thanks for the suggestion! We've added the reference genome sizes to Table 4 on page 19. Only

a few of the reference sequences were included for the substantial differences between the

sequence contiguity observed in the Ariadne and reference-based deconvolution with respect to

no deconvolution.

7. It would be informative to include SLR deconvolution results on more complex metagenomic

datasets than MOCK20, as there are publicly available datasets e.g. human gut sample.

Please see section 2.7 on page 6, where we have simulated datasets of 50 to 100 species.

8. The authors show that the reference deconvolution procedure results in a significantly higher

misassembly rate. Could this be alleviated by changing the maximum fragment length parameter

currently set at 200 kbp, thus obtaining a more accurate gold standard deconvolution?

This was something we'd be wondering about too! Especially with the variations in linked-read

fragmentation methods from sonication to enzyme-based digestion. The fragment length

parameter as we've conceived it- an upper bound on the distance between the upstream-most

and downstream-most reads in a single cloud- is an attempt at including the maximal number of

reads from a single reference sequence. In that spirit, we have varied it from 100 kbp to 400 kbp,

and found that the results are fairly similar in both cloud purity and assembly. We have also

noted in the text (middle of page 4) that the reference-based results are present as a high-water

comparison only. If the species composition of the original sample were already known,

downstream applications such as variant-calling or strain inference are probably better uses of

the data, as opposed to de novo assembly which is for species composition discovery.

1. Vertex v_j is not defined in the equation describing the forward Dijkstra graph.

Addressed on page 9. Vertex v_j is the 3’-terminal vertex of the edge that the read maps to.

2. Which dataset is used for Supplementary Figure 3?

Dataset specifications have been added to all figures in the supplementary materials.

• Supp. Figure 1: All 4 datasets indicated by column numbers

• Supp. Tables 1, 2: All 4 datasets indicated by the leftmost column

• Supp. Table 3: MOCK20 10x and TELL-Seq

• Supp. Table 4: MOCK5 LoopSeq and MOCK20 TELL-Seq

• Supp. Figure 2: MOCK20 TELL-Seq

• Supp. Table 5 and Fig. 3: 50- and 100-species simulated 10x datasets

• Supp. Tables 6 and 7: MOCK20 TELL-Seq

• Supp. Figures 4 and 5: Subset of MOCK5 10x

3. Page 10, Step2B: "This process is conducted via binary search of sorted vertex names in the

forward and reverse Dijkstra graphs," Sorted with respect to distance from start node? Overall,

this procedure could be described in more detail.

4. Page 10, Step2B: "if one of the following conditions holds" and "Else, if neither of the above is

true," I only see one condition in the paragraph.

5. Page 10, Step2B: Is it correct that only an upstream read (w.r.t. the assembly graph) can

identify downstream reads as part of the same fragment? That is, if the true genomic order of

reads in a read cloud is r_1, r_2, r_3, then r_1 can identify both r_2 and r_3 as belonging to the

same cloud, but r_2 can only identify r_3. Thus, if the algorithm starts with r_2 and identifies r_3

as belonging to the same fragment, when exploring r_1 as r_i, one only need to connect r_1 with

r_2. More details around this could be given.

We've cleaned up the wording of Step 2B (page 11) to clarify the mechanism behind find

connected components and get rid of the ungainly if-else structure. In short, for each read i, we

construct forward and reverse Djikstra graphs of neighbouring vertices with the maximum

distance away being the search distance (as defined in the manuscript). Then, every other read

j's vertices (i.e.: the two vertices bordering the edge the read aligns to) are compared to that

neighbour vertex set. If the other reads j's vertices are in the set, the two reads i and j are said to

be connected components. Since we're looking at both the forward (vertices downstream of the

edge that read i aligns to) as well as reverse (vertices upstream of the edge that read i aligns to)

Djikstra graphs, we can identify read j's that are connected both upstream and downstream of

read i.

In cloudSPAdes, the vertices are identified by integers, so sorting a set of vertices (i.e.: the

vertices found in read i's forward + reverse Djikstra graphs) is just a step to make subsequent

lookups of the other read's vertices (i.e.: potential shared vertices) easier and faster.

6. Page 10, section 5.2.3: There is no motivation given for described processing of enhanced

clouds composed of a single pair of reads. Nothing indicates that adding such clouds to the

smallest enhanced read cloud with the same UMI would result in a set of reads originating from

the same fragment if more than one non-trivial enhanced read cloud with the same UMI is

present.

This is correct! The methods was written for an older version of Ariadne. At time of submission,

the actual procedure we used was to add all 'disconnected' reads (i.e.: not connected

components of any other reads or unmappable to the assembly graph) to a separate read cloud

of their own. This sentence has been removed.

7. Page 11, Section 5.4: "We provide a short summary of the mathematical model that justifies

the modelling" In the text that follows, no mathematical model is given. Either change to "[23]

provided a mathematical model..." Or describe the model.

Citation added, thank you!

8. "We termed this method 'reference deconvolution' as it represents the maximum likelihood

inference of the genomic fragments" It is unclear what model the 'maximum likelihood

inference' is referring to, and I believe in such a case, it would rather be 'maximum likelihood

estimate'. Either a model should be provided, or it is easier to avoid the term' maximum

likelihood' here.

Thanks for the feedback on the wording! We've removed the term.

9. Table 4. Clarify the meaning of the asterisk, particularly "no associated NA50 ".

The intent was clarified in the table's caption (page 19). Basically, missing NA50 values mean that

the assembly did not have sufficient sequence material that was alignable to the reference

sequence for an NA50 to be calculated.

10. Section 2.3: "Furthermore, reference-based deconvolution generates a similar number of

clouds of a similar size to Ariadne". The MOCK20 10X number of clouds (3.1M vs 6.9M) stands

out significantly from this claim and should be discussed or at least mentioned.

11. Section 2.3: "The ideal deconvolution based on reference-mapped positions is shown in the

Figure 1 middle row, where there are extremely few clouds with P < 1." This cannot be inferred

from the dot-plot figure. For example, from MOCK20 there are many dots below 1, although I

am guessing the density of dots at 1 is much higher.

Figure 1: Previously, altair, the Python package we used to visualize the cloud size x cloud purity,

was prohibitively slow. Since we've replaced it with seaborn, we are now able to plot every cloud

in each of the datasets (in the 100,000s) instead of a down-sampled subset of 40,000. Hopefully,

this demonstrates the comparability of the gold-standard (reference-deconvolved) and Ariadne-

deconvolved clouds in terms of both size and compositional purity. You were also previously

correct- the density of dots at 1 is indeed much higher- and we've tried to decrease dot colour

saturation and size in order to more clearly reflect this.

Another aesthetic update we made was to harmonize the colour scheme with Figure 2, so that

the results can be more clearly matched up.

- Typo "SLR datsets" on page 7

- "Additionally, is an extension of the EMA approach that makes"

- "Chimeric contigs are hazardous for downstream analyses, since as the binning of contigs into

metagenomic-assembled genomes"

- "...we will be able to all reads with the same UMI..."

- "within the maximum search distance d the 3'-terminal"

All addressed, thank you!

Reviewer #2:

So my main suggestion is that the title, abstract, background, discussion, and conclusion clearly

identify the problem using terms like"microbiome" and "metagenome", not just "complex

genomic mixtures" and"complex samples", and do so up front.

Similarly, the first paragraph of the Discussion is a puzzling disconnectfrom the paper. I would

suggest making a general statement first that sumsup the results for readers who jump to this

part of the text to understandwhat the paper is trying to say.

This is really helpful advice. We've added more targeted phrasing to the abstract as well as the

beginning of the background and the discussion (see highlighted text in both sections), which

now also has a section on taxonomic classification, which should help to make the purpose of

our paper more clear-cut. The reason why we kept it general to begin with was because our

secondary focus was to present a comparison of different linked-read technologies from the

perspective of de novo assembly quality, which hasn't previously been done. The last paragraph

of the Discussion has been rightfully moved to the end, where it belongs with the rest of the

material on comparing linked-read technologies.

* p1 line ~48 second column, "When sequencing depth is low, the assembly graph contains

many ambiguous junctions comprised of sequence material from multiple genetically similar

species and/or strains." This is clearly true of high depth sequencing too - presumably the

authors mean it's harder to deconvolute at low depths? Or something else? Please clarify.

We've added a clarifying sentence after that one, namely- ‘Low sequencing coverage means that

some edges in the de novo assembly de Bruijn graph are interpreted as sequencing errors, and

mistakenly removed.’

* p2 line 32 colum 2, For "Many taxonomic lineages identified ... are not represented in

reference databases", I was surprised that Hug et al. was the only reference here. Maybe one or

another of the large scale studies (like the various Almeida papers) would be good to add here,

if there is room in the citations.

Thanks for the suggestion! We've added the Almeida et al. 2019 paper as well as one that

discusses the microbiome of a saline lake i.e.: an extreme environment on page 2.

* The second half of that paragraph is also poorly motivated, I think - reference based analyses

will not _bias_ towards the reference, they will _completely ignore_ anything not in the

reference. I am not sure exactly what the point of this sentence is? I feel like there are more

straightforward ways to make the case for de novo assembly.

We've added one more line at the end of the paragraph to explain why reference-based analyses

are not ideal for some samples to properly justify using a de novo assembly approach on page 2.

* p3 line 10 second column, a reference or word is missing after "Additionally."

We've moved the citation to the correct location at the front of the sentence.

* p3 line 33 second column - traditionally de Bruijn assembly graphs (presumably compact

DBGs?) do not contain linkage information. Although the ones produced by cloudSPADes might?

Please state if so.

The cloudSPAdes' cDBG formulation is actually the same as MetaSPAdes'. The assembly graph

itself is not constructed using UMI/3' barcode information. The only time at which UMI/3'

barcode information is applied is during the contig/scaffold resolution step i.e.: when the

algorithm tries to infer the order of long edges in the assembly graph using the similarity

between the set of barcode-sets associated with each edge, and then fill the gaps between long

edges with the shorter edges (paraphrased from the cloudSPAdes manuscript).

* p5 line 57 column 1 - "significantly positive differences" could be rephrased - do you mean

"positive differnces that were statistically significant" or "large differences that were positive"?

We have gotten rid of the word 'significantly' in the manuscript on page 5. Thanks!

**2^nd^ round**

**Reviewer 1**

The authors have done a good job with the revision and addressed most of my concerns. It is nice to see the substantial speedup and additional taxonomic assignments application.

I have one remaining significant remark regarding the new simulated datasets in the revision:

- The new simulated datasets are welcomed, but it is worrying to see that Ariadne-deconvoluted clouds drop remarkably in quality (over purity and higher entropy) and are nearly on par with non-de convoluted clouds (Supplementary Tab. 5). This is surprising to me, given the better results on biological data. The authors reason that the lower quality is because of the increase in the number of species. This prediction could be addressed by simulating an LRSim-simulated 10x dataset from the MOCK20 to check whether the claim holds. Of course, there may be something with the simulator assumptions or related to the search distance, but I think this result deserves further investigation.


Some additional minor comments:

- New analysis section 2.8: This section needs a brief explanation of the methodology behind the analysis. Please describe the setup of the experiment. For example, how is the 'promotion' computed? What tools are used to make the taxon classification?


- New analysis section 2.7: there are probably some missing/erroneous links to supplement tables/figures in this section. I would have expected Supp. Table 5 (and perhaps Supp Fig. 2) is to be referenced in this section. For example, "there is a sizable increase in the average purity of Ariadne-deconvolved clouds relative to non-deconvolved reads in the 50-species dataset (Supp. Table 4)." Should it be Supp. Tab 5?

**Reviewer 2**

In this paper, Mak et al. explore deconvolution of synthetic long read/linked read data sets (for maintaining haplotype/genome linkages across long distances) using assembly graphs.

This is a great update of the paper, addressing many of my concerns and confusions!

A few nits remain that could help with clarity - in my first review, I said:

* p1 line ~48 second column, "When sequencing depth is low, the assembly graph contains many ambiguous junctions comprised of sequence material from multiple genetically similar species and/or strains." This is clearly true of high depth sequencing too - presumably the authors mean it's harder to deconvolute at low depths? Or something else? Please clarify.

While the authors added a sentence to indicate that error trimming is the cause of downstream problems with low coverage, the statement is still quite odd and I think could be reworded. Maybe something like "Assembly graphs contain many ambiguous junctions ..., and at low coverage real sequence cannot be distinguished from erroneous sequence." I'm sorry to pick on it more but it really stands out to me as a confusing statement, as is!

p2 column 2 line 18 contains a justification of de novo assembly for metagenomics. I like it! But I don't think it belongs at the bottom of the paragraph it's in? It seems to be the first mention of the word "reference" and, if kept, should be in the first 2-3 paragraphs I think.
(I referenced this in my first review and upon re-reading it I think it's just in the wrong place in the paper.)

I offer these suggestions to the authors as a way to improve an already excellent paper. I don't need to see another revision.

**Authors’ response**

Reviewer #1:

The new simulated datasets are welcomed, but it is worrying to see that Ariadne- deconvoluted clouds drop remarkably in quality (over purity and higher entropy) and are nearly on par with non-de convoluted clouds (Supplementary Tab. 5). This is surprising to me, given the better results on biological data. The authors reason that the lower quality is because of the increase in the number of species. This prediction could be addressed by simulating an LRSim-simulated 10x dataset from the MOCK20 to check whether the claim holds. Of course, there may be something with the simulator assumptions or related to the search distance, but I think this result deserves further investigation.

Thank you for the suggestion. Using the same LRSim/LongRanger steps, we simulated a 10x sequencing dataset from the MOCK20 reference genomes, and added the deconvolution and de novo assembly results to Supplementary Table 5 and Supplementary Figure 3, as well as mentions of the new simulated dataset to the second column on page 6.

When comparing read cloud summary statistics in Table 3 to Supp. Table 5, we can see that there are fewer read clouds in the real 10x dataset (503,205 vs. 1,720,220), the read clouds are generally larger (186 vs. 58.13 reads on average per cloud) and more pure (0.38 vs. 0.24). These differences are probably due to mismatches between the default LRSim parameters, which are estimates of wet laboratory library prep outcomes, and the real 10x sequencing library's properties.

Nevertheless, the summary statistics and assembly results support our earlier hypothesis that deconvolution quality is highly dependent on the number of species in the originating sample. While the average purity of Ariadne-deconvolved clouds is slightly lower than the gold-standard clouds in the 20-species simulated dataset, it is less than half in the 50- and 100-species datasets (Supp. Table 5 column 3). The size of the Ariadne-deconvolved clouds is also much closer to that of the gold-standard clouds in the 20-species simulated dataset, whereas Ariadne did not reduce the size of the raw read clouds much in the 50- and 100-species datasets. Similarly, the average contiguity of the Ariadne-deconvolved assembly is higher than without deconvolution (Supp. Figure 3A and Figure 2 column 3), but there isn't much of a change in the largest alignment and the relative misassembly rate (Supp. Figure 3B and C).

New analysis section 2.8: This section needs a brief explanation of the methodology behind the analysis. Please describe the setup of the experiment. For example, how is the 'promotion' computed? What tools are used to make the taxon classification?

We've added a section explaining the taxonomic classification promotion algorithm to the end of the Methods (see 5.5 on page 12). In brief, reads at higher taxonomic ranks (ex. kingdom, phylum) are 'promoted' to lower ranks (ex. family) if the rest of the reads in that same read cloud are consistently classified to a taxon at that lower rank. In the Methods section, 2/7 read classifications are improved to the family level because the other 5/7 reads are all classified to the same family taxon.

- New analysis section 2.7: there are probably some missing/erroneous links to supplement tables/figures in this section. I would have expected Supp. Table 5 (and perhaps Supp Fig. 2) is to be referenced in this section. For example, "there is a sizable increase in the average purity of Ariadne-deconvolved clouds relative to non- deconvolved reads in the 50-species dataset (Supp. Table 4)." Should it be Supp. Tab 5?

Thanks for pointing that out! 'Supp. Table 4' was indeed supposed to be Supp. Table 5. We've also more explicitly indicated to the reader to compare Supp. Table 2 (summary statistics from the mock metagenome datasets) to Supp. Table 5 (summary statistics from the fully simulated datasets). See the changes on the middle right of page 6.

Reviewer #2:

* p1 line ~48 second column, "When sequencing depth is low, the assembly graph contains many ambiguous junctions comprised of sequence material from multiple genetically similar species and/or strains." This is clearly true of high depth sequencing too - presumably the authors mean it's harder to deconvolute at low depths? Or something else? Please clarify.

- While the authors added a sentence to indicate that error trimming is the cause of downstream problems with low coverage, the statement is still quite odd and I think could be reworded. Maybe something like **"Assembly graphs contain many ambiguous junctions ..., and at low coverage real sequence cannot be distinguished from erroneous sequence."** I'm sorry to pick on it more but it really stands out to me as a confusing statement, as is!

Thank you for your suggestions! We've merged that poorly worded sentence and the sentence immediately after it to hopefully make the point (which you've correctly interpreted) less confusing!

* p2 column 2 line 18 contains a justification of de novo assembly for metagenomics. I like it! But I don't think it belongs at the bottom of the paragraph it's in? It seems to be the first mention of the word "reference" and, if kept, should be in the first 2-3 paragraphs I think. (I referenced this in my first review and upon re-reading it I think it's just in the wrong place in the paper.)

This is a good point! Upon rereading the section and the surrounding text, we've simply moved down so that section, which justifies why assembly is an important part of metagenomic analysis, is now discussed with the rest of the metagenomics material.

**3^rd^ round**

**Reviewer 1**

The authors have addressed my concerns. It is good to see that the added experiment matched their assumption and that proper description for the promotion experiment has been added.


Typos:

- "When comparing read cloud summary statistics in of the real to the simulated 20-species datsets"

- "6 k_0" -> "7 k_0"
